# *Trichoderma*-Induced Ethylene Responsive Factor MsERF105 Mediates Defense Responses in *Malus sieversii*

**DOI:** 10.3389/fpls.2021.708010

**Published:** 2021-10-29

**Authors:** Shida Ji, Zhihua Liu, Yucheng Wang

**Affiliations:** ^1^Key Laboratory of Biogeography and Bioresource in Arid Land, Xinjiang Institute of Ecology and Geography, Chinese Academy of Sciences, Ürümqi, China; ^2^University of Chinese Academy of Sciences, Beijing, China; ^3^College of Forestry, Shenyang Agricultural University, Shenyang, China; ^4^State Key Laboratory of Tree Genetics and Breeding, Northeast Forestry University, Harbin, China

**Keywords:** *Trichoderma* biofertilizer, *Malus sieversii*, transcriptome, plant hormone, *Alternaria*

## Abstract

*Trichoderma* can induce plant hormone signal pathways mediating plant defenses, resulting in broad-spectrum resistance to phytopathogens. Herein, *Malus sieversii* seedlings were treated with *Trichoderma* biofertilizer and/or *Alternaria alternata* f. sp. *mali*, and transcriptome analysis revealed significant differential expression. There was a high similarity between the transcriptome expression profiles of *Trichoderma*-induced and *A. alternata*-infected *M. sieversii* samples for genes related to jasmonic acid (JA), ethylene, and salicylic acid (SA) signaling pathways. Additionally, *Trichoderma* biofertilizer activated numerous disease-resistant genes (*ERF*, *NAC*, *bHLH*, and *STK*) and defense response genes (*DRP*, *ABC*, and *HSP*). Among transcription factors, members of the ERF family were the most differentially expressed (18 *ERF*s), indicating that they may be closely related to defense responses. Among ERFs, differential expression of *MsERF105* was the most significant (upregulated 27.6-fold compared to controls). *MsERF105* was heterologously expressed in PdPap poplar (*Populus davidiana* × *Populus alba* var. *pyramidalis* Louche), and following infection with *A. alternata* (Aal), transgenic PdPap-MsERF105s plants displayed lower malondialdehyde (downregulated 41.4%) and reactive oxygen species (ROSs) levels, and higher reductase activities, especially superoxide dismutase (SOD; upregulated 77.5% compared to PdPap-ROK2 plants). Furthermore, the lesion areas of PdPap-MsERF105s leaves were significantly smaller (0.2%) than those of PdPap-ROK2 leaves (∼26.0%), and the cell membrane integrity was superior for PdPap-MsERF105s leaves. Thus, *MsERF105* enhanced the resistance of PaPap poplar to Aal, presumably because *MsERF105* activates the expression of *PR1* and *PDF1.2*. In conclusion, *Trichoderma* biofertilizer modulated the differential expression of numerous disease resistance genes and defense response genes in *M. sieversii* in response to pathogen attack, and *MsERF105* played important roles in this process.

## Introduction

Trichoderma can penetrate and colonize plant roots, triggering plant defense responses, and is commonly used as an inducer of plant defenses against pathogens (Poveda, 2021). The presence of fungal prey and the availability of root-derived nutrients may have been major attractors for the ancestors of *Trichoderma* to establish themselves in the rhizosphere and thereby facilitate the evolution of positive interactions with plants (Druzhinina et al., 2011). In *Trichoderma*-root interactions, *Trichoderma* secretes molecules that trigger induced systemic resistance, such as xylanases, peptaibols, swollenin, and cerato-platanins (Rosa et al., 2012). Eliciting plant response protein 11 (Epl1) secreted by *Trichoderma formosa* triggers immune responses against *Tomato mosaic virus* infection in *Nicotiana benthamiana*, and against *Alternaria brassicicola* infection in *Brassica rapa* subsp. *chinensis*, by activating genes (*JAZ1*, *JAR1.2*, *CALS1*, *NPR1*, *PAL1*, and *EDS1*) related to defense responses (Cheng et al., 2018). Additionally, 6-pentyl-alpha-pyrone from *Trichoderma koningii* induces systemic resistance in tobacco against tobacco mosaic virus (TMV), stimulates the activities of pathogenesis-related (PR) enzymes [superoxide dismutase (SOD), peroxidase (POD), and polyphenol oxidase), and upregulates the expression of defense-related genes (PR-a, PR-b, and PR-10) (Taha et al., 2021).

In recent years, numerous studies have contributed to uncovering the molecular basis of the beneficial effects of *Trichoderma* on plants. *Trichoderma*-induced plant transcriptome expression profiles display high similarity to those induced by pathogen infection (Cheng et al., 2018). Furthermore, *Trichoderma*-induced plant resistance against pathogens is mainly mediated by jasmonic acid (JA), ethylene (ET), and salicylic acid (SA) signaling pathways (Gelsomina et al., 2018). Inoculation with *Trichoderma asperellum* WKSSO2-4-18 strongly induced six defense-related genes in soybean seedlings, namely, those involved in the SA pathway (endoglucanase and chalcone synthase) and the JA/ET pathway (chitinase, defensin precursor, allene oxide synthase, and basic POD genes) (Pimentel et al., 2020). In addition, in tomato induced by *Trichoderma erinaceum*, *SlWRKY31* and *SlWRKY37* are upregulated, whereas *SlWRKY4* is downregulated, resulting in increased resistance to the vascular wilt pathogen *Fusarium oxysporum* f. sp. *lycopersici* (Aamir et al., 2019). Plants treated with *Trichoderma harzianum* T22 displayed overexpression of transcripts encoding several families of defense-related transcription factors (bZIP, MYB, NAC, ERF, and WRKY), suggesting that the fungus contributes to the priming of plant responses (Coppola et al., 2019). Besides, in our previous, it is found that *Trichoderma rossicum* T7 (TroT7) and *T. harzianum* T2 (ThaT2) can enhance the resistance of *Malus sieversii* to *F. oxysporum* and *Alternaria alternata* f. sp. *mali* (Aalm) (Fang et al., 2019). Thus, *Trichoderma* can trigger plant defense responses in different pathways.

ERF transcription factors are the largest branch of the AP2/ERF transcription factor superfamily, which is characterized by a conserved AP2 binding domain of 57–66 amino acids (Huang et al., 2020). The AP2/ERF domain contains two conserved elements, namely, YRG and RAYD (Okamuro et al., 1997). *ERF* genes are involved in responses to biotic stresses through plant hormone signaling pathways (Caarls et al., 2016). Similarly, soybean GmERF3 transgenic tobacco can respond to the regulation of many plant hormones, such as SA, JA, and ET (Zhang et al., 2009). In addition, in transgenic tobacco, the transcription factor *GmERF3* activated some PR genes expression, and improved the resistance of tobacco to *Ralstonia solanacearum*, *A. alternata* (Aal), and TMV (Zhang et al., 2009). Overexpression of *ZmERF105* can improve the resistance of maize to northern leaf blight (*Exserohilum turcicum*), while the erf105 mutant showed the opposite phenotype. In addition, the activities of SOD and POD in ZmERF105 overexpression lines were significantly higher than those in wild-type plants (Zang et al., 2020). Similarly, overexpression of ZmERF105 enhanced the expression of PR genes such as *ZmPR1a*, *ZmPR2*, *ZmPR5*, *ZmPR10.1*, and *ZmPR10.2* (Zang et al., 2020). AtERF96 overexpression enhances the resistance of *Arabidopsis thaliana* to necrotic pathogens such as *Botrytis cinerea* and *Pectobacterium carotovorum* by upregulating the expression of *PDF1.2a*, *PR-3*, *PR-4*, and *ORA59* (Catinot et al., 2015).

In the present study, *M. sieversii* seedlings were treated with *Trichoderma* biofertilizer or Aalm, and differentially expressed genes (DEGs) were analyzed by RNA sequencing (RNA-Seq). Gene ontology (GO) function classification, Clusters of Orthologous Groups (COG) function classification, and Kyoto Encyclopedia of Genes and Genomes (KEGG) classification analyses were conducted. In addition, differential expression of transcription factors was explored. The *M. sieversii MsERF105* gene was heterologously expressed in *Populus davidiana* × *Populus alba* var. *pyramidalis* Louche (PdPap poplar), and PdPap seedlings were infected with Aal. The antioxidant ability of transgenic PdPap-MsERF105s plants was evaluated by determining malondialdehyde (MDA) and reactive oxygen species (ROSs) content, reductase (SOD, POD, and CAT) activities, resistance to Aal, relative lesion area, and cell membrane permeability. Differential expression of defense response genes (*PR1* and *PDF1.2*) was assessed. Based on the above results, the functions of ERFs in defense responses in *M. sieversii* were evaluated.

## Materials and Methods

### Plant Material and Strains

*Malus sieversii* seeds from a Xinjiang wild fruit forest were germinated in the laboratory (temperature 28°C and humidity 70%) to obtain 2-month-old seedlings. Apple-spotted defoliation pathogen Aalm was obtained from the Key Laboratory of Biogeography and Bioresource in Arid Land, Xinjiang Institute of Ecology and Geography. Poplar leaf blight pathogen Aal was obtained from the State Key Laboratory of Tree Genetics and Breeding (Northeast Forestry University, China). *Agrobacterium tumefaciens* EHA105 and the pROK2 vector were used for the genetic transformation of PdPap poplar. PdPap poplar seedlings were cultured aseptically in a liquid rooting medium at 25°C (Ji et al., 2017).

### Preparation of *Trichoderma* Biofertilizer

Spores of TroT7 and ThaT2 were separately inoculated into potato dextrose agar (PDA) medium and cultured at 28°C for 1 week. Both *Trichoderma* strains were identified in a previous study (Ji et al., 2021). Spores were collected and diluted to 10^8^ spores/ml. The number of spores was counted using a blood cell counting board. Next, 10 ml of spore suspension was added to 300 g solid fermentation medium (thickness 1–1.5 cm) and mixed. The solid fermentation medium for *Trichoderma* comprised rice husk (19.9% w/w), wheat bran (5% w/w), (NH_4_)_2_SO_4_ (0.1% w/w), and H_2_O (75% w/w). The tray was then sealed with preservative film. TroT7 and ThaT2 were cultured at 28°C for 1 week, spores of both *Trichoderma* strains were mixed (1:1) as *Trichoderma* biofertilizer, and the concentration of conidia in the biofertilizer was 10^8^ spores/ml.

### Collection of Samples for RNA-Seq

Two-month-old *M. sieversii* seedlings were subjected to four different treatments, three biological replicates each treatment were set. The samples treated without *Trichodema* biofertilizer and Aalm, as negative control, were collected at 0 h, the other samples were collected at 6 and 48 h post-induction (hpi), and all samples were stored at −80°C for RNA-Seq. For the Tri treatment, seedlings were irrigated with 50 ml *Trichodema* biofertilizer (10^8^ spores/ml), one leaf of each seedling was stabbed, and inoculated without Aalm mycelium. For the Aalm treatment, seedlings were irrigated with 50 ml sterilized water, and one leaf of each seedling was stabbed and inoculated with 0.6 cm disks containing Aalm mycelium, which was cultured in PDA medium at 28°C for 10 days, and 0.6 cm disks were cut with a hole punch. For the Tri + Aalm treatment, seedlings were irrigated with 50 ml *Trichodema* biofertilizer (10^8^ spores/ml), and one leaf of each seedling was stabbed and inoculated with 0.6 cm disks containing Aalm mycelium. For the negative control, seedlings were irrigated with 50 ml sterilized water, one leaf of each seedling was stabbed and inoculated without Aalm mycelium.

### RNA-Seq

Total RNA was extracted using the cetyltrimethylammonium bromide (CTAB) method (Jiang and Zhang, 2003), following analysis of RNA quality using a NanoDrop 2000C instrument (Thermo, Germany), mRNA was enriched using Oligo (dT) magnetic beads and randomly interrupted. Using mRNA as a template, the first and second cDNA strands were synthesized with random hexamers, then purified double-stranded cDNA was end-repaired with T4 DNA Polymerase (Promega, Madison, WI, United States) by inoculation at 37°C for 30 min, and a poly-A tail was attached to the sequencing connector. Finally, the cDNA library was obtained by PCR enrichment (Aird et al., 2011). High-throughput sequencing of RNA-Seq samples was performed by Illumina hiseq (Solexa, United Kingdom) after the library was tested with an Agilent 2100 instrument (Agilent, Palo Alto, CA, United States).

Based on sequencing by synthesis technology, an Illumina hiseq high-throughput sequencing platform was used to sequence the cDNA library to produce a large number of high-quality reads (raw data). This was filtered to remove joined and low-quality reads to obtain high-quality clean data, which was assembled using Trinity software (Grabherr et al., 2011). Then, GO, COG, and KEGG classifications were conducted according to the previous studies (Ashburner et al., 2000; Ogata et al., 2000; Tatusov et al., 2001).

### Expression of Genes Involved in Plant Hormone Signal Pathway Using RT-qPCR

Following the three treatments (Tri, Aalm, and Tri + Aalm) at 28°C and 70% humidity, the treatment of samples was the same as that described for RNA-Seq, three plants (biological replicates) each treatment were set. Leaf samples were collected at 6 and 48 hpi, and untreated seedlings served as controls, leaf samples were collected at 0 hpi. Total RNA was extracted using the CTAB method, and reverse-transcribed into cDNA for quantitative real-time polymerase chain reaction (RT-qPCR). The gene-encoding *MsActin* (MZ605395) in *M. sieversii* was used as an internal reference. Three technical replicates were performed for each cDNA sample. Primers for RT-qPCR (Supplementary Table 1) were designed by using Primer Premier 6.0 software (PREMIER Biosoft, San Francisco, CA, United States).

### Expression of ERF Family Genes Using RT-qPCR

The method is the same as given in the section “Expression of genes involved in plant hormone signal pathway using RT-qPCR.” Following the three treatments (Tri, Aalm, and Tri + Aalm) at 28°C and 70% humidity, three plants (biological replicates) each treatment were set. Leaf samples were collected at 0, 3, 6, 12, and 24 hpi, and untreated seedlings served as controls. Three technical replicates were performed for each cDNA sample. Primers for RT-qPCR (Supplementary Table 1) were designed by using Primer Premier 6.0 software (PREMIER Biosoft, San Francisco, CA, United States).

### Construction of the Plant Expression Vector pROK2-MsERF105 and Poplar Transformation

The *MsERF105* gene was cloned by PCR using a sense primer (5′-ATCGTCTAGAGAGTTTCTAAATATGGCATCAGAAGC-3′) containing an *Xba*I (Promega, WI, United States) site (underlined) and an antisense primer (5′-CGATGGTACCTCAAATAACCATGAGCGGAGGATATC-3′) containing a *Kpn*I (Promega, United States) site (underlined). The resulting *MsERF105* PCR product and the pROK2 vector were double digested with *Xba*I and *Kpn*I at 37°C for 4 h, and ligated using T4 DNA ligase (Promega, United States) at 4°C for 12 h, to generate the pROK2-MsERF105 construct. *MsERF105* was transferred into PdPap poplar using the *A. tumefaciens*-mediated transformation system, and empty vector pROK2 was transferred in PdPap to generate the PdPap-ROK2 control. The method was described in detail in our previous study (Ji et al., 2016).

### The Resistance of Transgenic PdPap-MsERFs Plants to Aal

Aal was cultured in a PDA medium at 28°C for 10 days, and 0.6 cm disks were cut with a hole punch. Leaves from PdPap-MsERF105s and the control PdPap-ROK2 plants were stabbed with a needle, covered with 0.6 cm disks, and cultured at 28°C for 5 days, three biological replicates each treatment were set. The relative area of disease spots was calculated by the Chalkiness 1.0 program (China). First, infected leaves were scanned to obtain digital images, the software accurately determined the location and profile of disease spots, and the relative area of disease spots was evaluated (Zheng and Wu, 2008).

### Determination of MDA Content and Reductase Activity

Following treatment of PdPap-MsERF105s and the control PdPap-ROK2 plants with 0.6 cm disks containing Aal mycelia, leaves were collected at 0, 3, 6, 12, 24, 48, and 72 hpi, 0.1 g was weighed using an analytical balance, ground in a pestle and mortar with liquid nitrogen, and the leaf powder was added to extraction solution (from the kit mentioned below) and centrifuged at 1,100 × *g* for 10 min, three biological replicates each treatment were set. The supernatant was used to determine the MDA content using an MDA-2-Y kit (Suzhou Comin Biotechnology Co., Ltd., china). The activities of SOD, POD, and catalase (CAT) were determined using dedicated kits (POD = A084-3; SOD = A001-1; CAT = A007-1; Nanjing Jiancheng Bioengineering Institute, China), three biological replicates each treatment were set.

### Leaf Staining for ROS Quantification and Plant Membrane Integrity

Evans blue and nitrotetrazolium blue chloride (NBT) were dissolved in 10 mM phosphate-buffered saline (pH 7.8), and diaminobenzidine (DAB) were dissolved in 50 mM Tris-acetate buffer (pH 5.0) (Hernández et al., 2001), and the final concentration of the staining solution was 1 mg/ml. NBT and DAB staining solutions were used immediately after preparation. Following treatment of PdPap-MsERF105s and the control PdPap-ROK2 plants with 0.6 cm disks containing Aal mycelia for 3 days, leaves were cut and stained with each of the three staining solutions as described in our previous study (Ji et al., 2021), and 10 biological replicates of each treatment were set.

### Differential Expression of Defense Response Genes in PdPap-MsERFs Plants

Following treatment of PdPap-MsERF105s and the control PdPap-ROK2 plants with 0.6 cm disks containing Aal mycelia, leaves were collected at 0, 3, 6, 12, 24, 48, and 72 hpi, and the expression levels of two *PR1* genes and two *PDF1.2* genes were determined by RT-qPCR, three biological replicates and technical replicates each treatment were set, respectively. The gene encoding *PdActin* in PdPap poplar served as an internal reference. The method was the same as described above for RT-qPCR analysis of ERF family genes. All primers are listed in Supplementary Table 1.

### Statistical Analysis

Statistical analysis was performed using SPSS 17.0 (IBM Corp., Armonk, NY, United States). Data are expressed as the mean of three or more independent replicates ± SD. Analysis of variance was conducted using Duncan’s test, and *p* ≤ 0.05 was considered significant.

## Results

### Differential Expression Following Treatment of *M. sieversii* With *Trichoderma* Biofertilizer and/or Aalm

**FIGURE 1:**
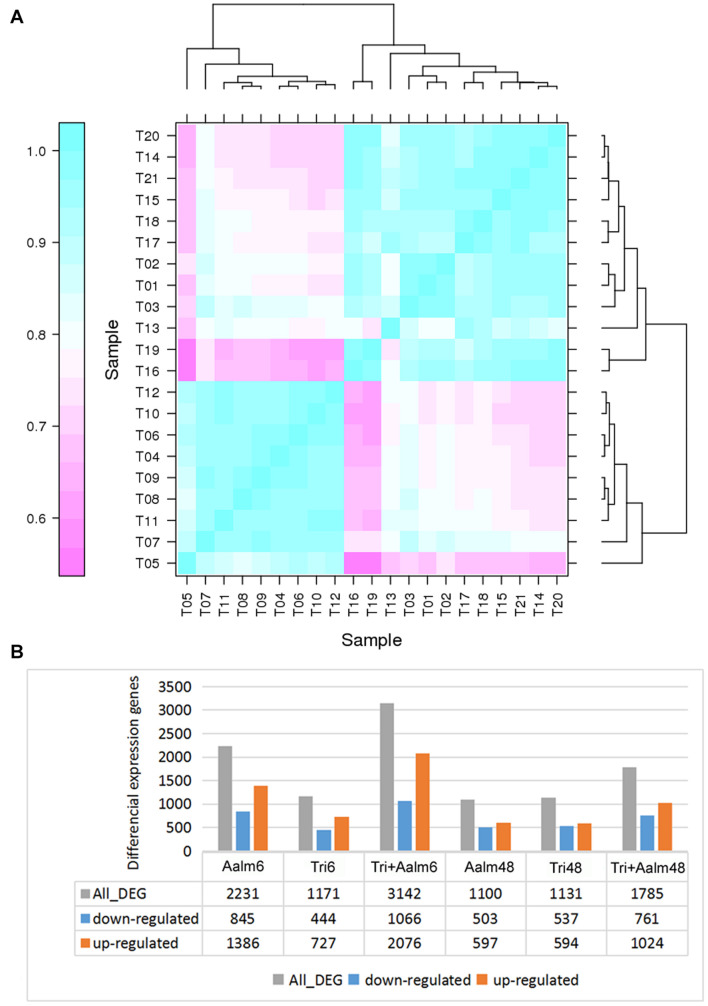
The differential expression genes of *Malus sieversii* treated with *Trichoderma* biofertilizer and Aalm. **(A)** The correlation analysis of gene expression pattern. T1-3 (Con): the control; T4-6 (Aalm6): *M. sieversii* treated with *A. alternata* f. sp. *mali* for 6 h; T7-9 (Tri6): *M. sieversii* treated with *Trichoderma* biofertilizer for 6 h; T10-12 (Tri + Aalm6): *M. sieversii* treated with *Trichoderma* biofertilizer and *A. alternata* f. sp. *mali* for 6 h. T13-15 (Aalm48): *M. sieversii* treated with *A. alternata* f. sp. *mali* for 48 h; T16-18 (Tri48): *M. sieversii* treated with *Trichoderma* biofertilizer for 48 h; and T19-21 (Tri + Aalm48): *M. sieversii* treated with *Trichoderma* biofertilizer and *A. alternata* f. sp. *mali* for 48 h. **(B)** The number of differential expression genes.

Following treatment with *Trichoderma* and/or Aalm, the gene expression pattern of *M. sieversii* was significantly different from that of controls, and the three replicate treatments yielded highly similar results, especially the three control replicates, which shared > 98% similarity (Figure 1A and Supplementary Table 2). In addition, the gene expression patterns of *M. sieversii* treated with *Trichoderma* biofertilizer and/or Aalm at the same timepoint were also similar (Figure 1A). Furthermore, the number of differential genes in *M. sieversii* treated with *Trichoderma* biofertilizer or Aalm was less than that following treatment with both, and more genes were upregulated than downregulated under all treatment conditions (Figure 1B).

### *Trichoderma* Biofertilizer Induces Plant Hormone Signal Transduction Pathways in *M. sieversii*

**FIGURE 2:**
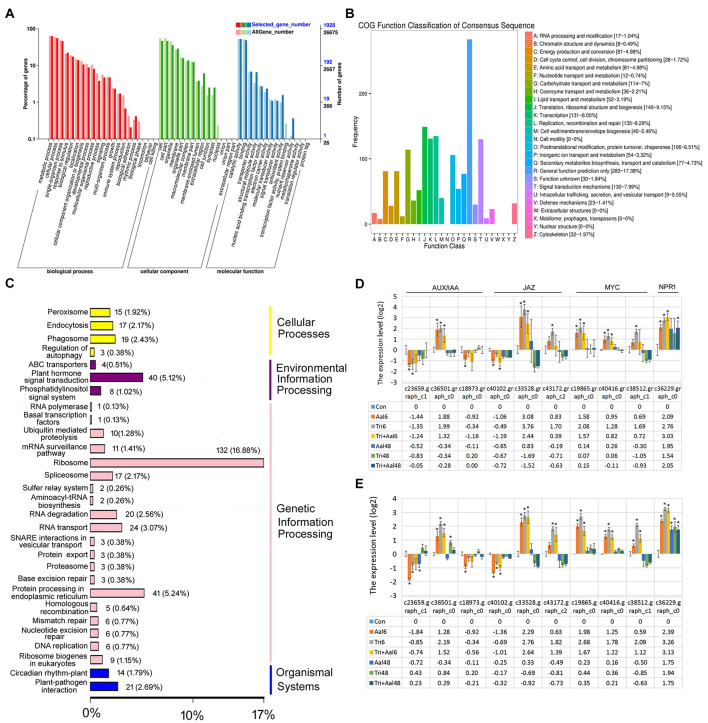
*Trichoderma* biofertilizer activates the plant hormone signal transduction pathways. **(A)** GO function analysis after *M. sieversii* treated with *Trichoderma* fertilizer for 6 h. **(B)** COG function classification of consensus sequence after *M. sieversii* treated with *Trichoderma* fertilizer for 6 h. **(C)** KEGG classification analysis after *M. sieversii* treated with *Trichoderma* fertilizer for 6 h. **(D)** DEGs involved in AUX/IAA, JA, and SA, after *M. sieversii* treated with *Trichoderma* fertilizer for 6 h. **(E)** Verify expression of genes in panel **(D)** using RT-qPCR. The data were counted by RT-qPCR, ANOVA was conducted using Duncan’s method, and *p* ≤ 0.05 was considered significant. The gene sequences have been deposited in GenBank, c23659.graph_c1 (MZ736861), c36501.graph_c0 (MZ736862), c18973.graph_c0 (MZ736863), c40102.graph_c0 (MZ736864), c33528.graph_c0 (MZ736865), c43172.graph_c2 (MZ736866), c19865.graph_c0 (MZ736867), c40416.graph_c0 (MZ736868), c38512.graph_c1 (MZ736869), and c36229.graph_c0 (MZ736870). COG: Clusters of Orthologous Groups, DEGs: differentially expressed genes, GO: gene ontology, JA: jasmonic acid, KEGG: Kyoto Encyclopedia of Genes and Genomes, RT-qPCR: quantitative real-time PCR, SA: salicylic acid.

Following treatment of *M. sieversii* with *Trichoderma* biofertilizer, GO function analysis showed that the identified DEGs were linked to response to stimulus, immune system process, and signal transducer activity categories (Figure 2A). Meanwhile, COG function classification also indicated the DEGs were closely related to signal transduction mechanisms (130 DEGs, 7.99% of all COG annotation genes) and defense mechanisms (23 DEGs, 1.41% of all COG annotation genes; Figure 2B). Finally, KEGG classification analysis indicated that the DEGs were involved in plant hormone signal transduction (40 DEGs, 5.12%) and plant-pathogen interactions (21 DEGs, 2.69%; Figure 2C), such as Auxin/indole3-3 acetic acid (AUX/IAA), JA, and SA signal transduction pathways. Following treatment of *M. sieversii* with *Trichoderma* biofertilizer and/or Aalm, the expression patterns of related DEGs were relatively similar to each other. Compared with Aalm treatment, *Trichoderma* biofertilizer induced higher expression levels (Figures 2D,E), which indicates that *Trichoderma* biofertilizer may activate JA and SA signal transduction pathways by upregulating the expression of *MYC2* and *NPR1*.

### Differentially Expressed Genes Related to Defense Responses in *M. sieversii*

**FIGURE 3:**
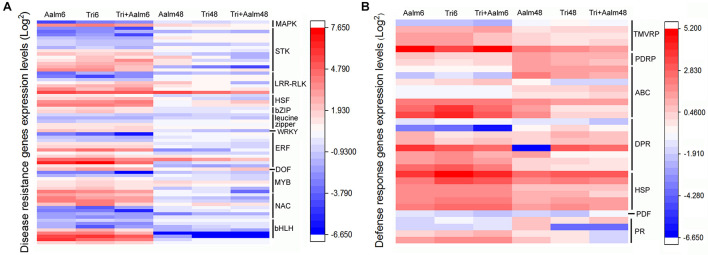
Different expression of resistant genes and defense response genes. **(A)** Differential expression of resistant genes. *MAPK*: mitogen-activated protein kinase gene, *STK*: serine/threonine-protein kinase gene, *LRR-RLK*: leucine-rich repeat receptor-like kinase gene, *HSF*: heat shock transcription factor gene, *bZIP*: bZIP transcription factor gene, *WRKY*: WRKY transcription factor gene, *ERF*: ethylene-responsive transcription factor gene, *DOF*: DNA binding with one finger gene, *MYB*: MYB transcription factor gene, *NAC*: NAC transcription factor gene, *bHLH*: bHLH transcription factor gene. **(B)** Differential expression of defense response genes. *PDRP*: pleiotropic drug resistance protein gene, *TMVRP*: tobacco mosaic virus (TMV) resistant protein gene, *ABC*: ABC transport protein gene, *DRP*: disease resistance protein gene, *HSP*: heat shock protein gene, *PDF*: defensin gene, *PR*: pathogenesis-related protein gene.

Following treatment of *M. sieversii* with *Trichoderma* biofertilizer and/or Aalm, expression levels of numerous resistance genes and defense response genes were significantly different (Figure 3). Resistance genes included *STK*, *LRR-RLK*, *AP2/ERF*, *NAC*, *bHLH*, *MYB*, and *HSF*. Differences in expression of these resistance genes were more significant after treating *M. sieversii* for 6 h than 48 h (Figure 3A). *AP2/ERF*, *NAC*, *bHLH*, *MYB*, *bZIP*, and leucine zipper genes were upregulated and downregulated after treatment for 6 h. These transcription factors can positively or negatively regulate downstream gene expression. Defense response genes included *DPR*, *ABC*, *HSP*, and *PR*, and the expression levels of the defense response genes were significantly upregulated after treatment for 6 and 48 h (Figure 3B). Thus, following treatment with *Trichoderma* biofertilizer and Aalm, expression levels of transcription factors were altered, and defense response genes were activated in response to pathogen attack.

### Differential Expression of ERF Transcription Factor Family Genes

**FIGURE 4:**
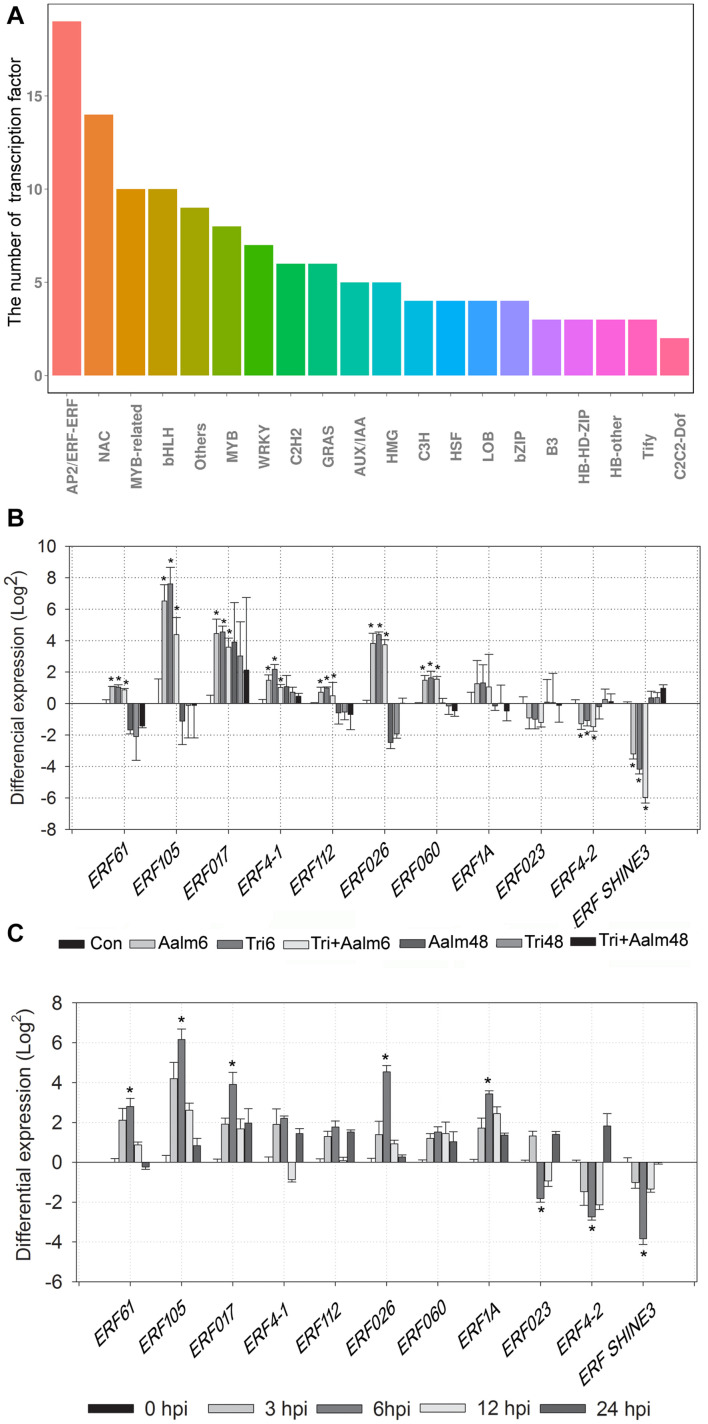
Differential expression of 11 genes of ERF transcription factor family. **(A)** The number of differentially expressed transcription factors from different families, after *M. sieversii* treated with *Trichoderma* biofertilizer for 6 h. **(B)** Differential expression of ERF transcription factor family genes, the data were counted from *M. sieversii* transcriptome data. **(C)** Differential expression of ERF transcription factor family genes, after *M. sieversii* treated with *Trichoderma* biofertilizer, the data were counted by RT-qPCR, ANOVA was conducted using Duncan’s method, and *p* ≤ 0.05 was considered significant. RT-qPCR: quantitative real-time PCR.

ERF transcription factors are closely related to plant defense responses. Following treatment with *Trichoderma* biofertilizer for 6 h, the number of deferentially expressed genes belonging to the AP2/ERF superfamily was more than other transcription factor families, including total of 19 AP2/ERF superfamily genes (Figure 4A). We, therefore, speculated that ERF family genes are widely involved in responding to pathogen attack in *M. sieversii*. The transcriptome data revealed that 11 *ERF* genes were differentially expressed, and expression levels of *MsERF105* were the highest among ERF family genes (Figure 4B). RT-qPCR analysis of ERF family genes also confirmed that *Trichoderma* biofertilizer strongly induced *ERF* genes expression, but mostly the expression of *MsERF105* (Figure 4C). Thus, *MsERF105* was selected for heterologous expression in transgenic PdPap plants to assess gene function.

### Antioxidant Ability of Transgenic PdPap-MsERF105 Plants

**FIGURE 5:**
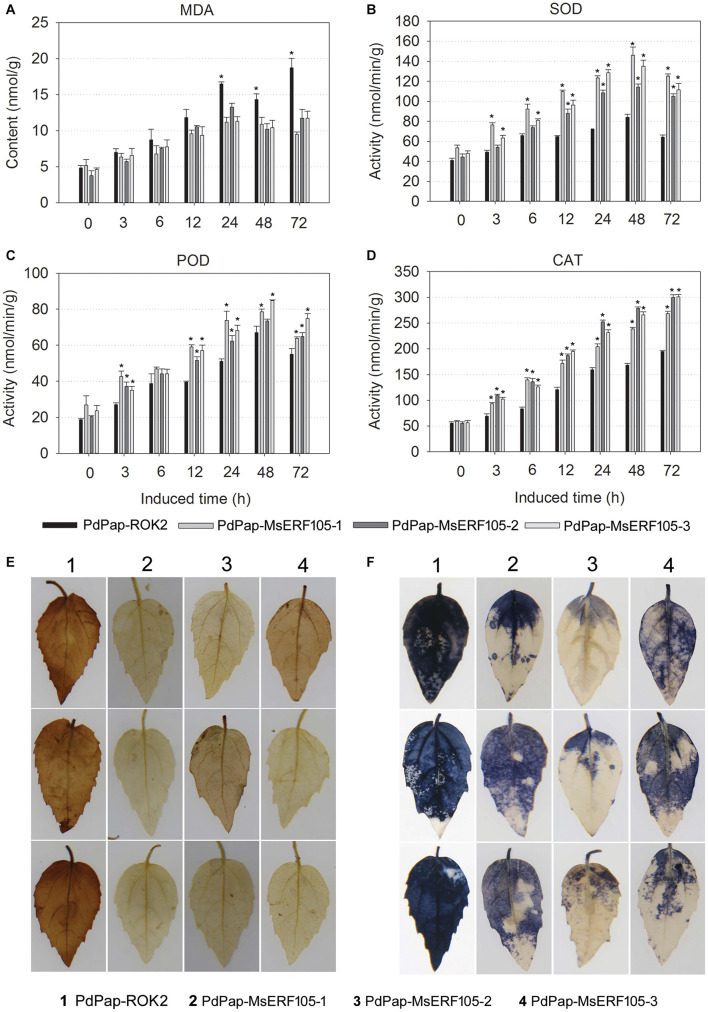
Antioxidant ability of PdPap-MsERF105. **(A)** The MDA content. **(B)** The SOD activity. **(C)** The POD activity. **(D)** The CAT activity. **(E)** DAB staining of PdPap leaves. **(F)** NBT staining of PdPap leaves. ANOVA was conducted using Duncan’s method, and *p* ≤ 0.05 was considered significant. CAT: catalase, DAB: diaminobenzidine, MDA: malondialdehyde, NBT: nitrotetrazolium blue chloride, POD: peroxidase, SOD: superoxide dismutase.

Pathogen attack can cause an elevation in ROS, resulting in severe damage to plants. Herein, transgenic PdPap-MsERF105s plants were treated with Aal, and the MDA content (<11.7 nmol/g at 72 hpi) was significantly lower than that of controls (18.7 nmol/g at 72 hpi) after 24 hpi, but the MDA content was not significantly different between PdPap-MsERF105s and control plants before 12 hpi (Figure 5A).

Furthermore, the activities of SOD, POD, and CAT were determined. The results showed that following treatment with Aal, the activities of SOD and CAT were significantly increased in PdPap-MsERF105s plants compared to controls after 6 hpi, and SOD activity peaked at 48 hpi. Additionally, values were > 105.1 nmol/min/g for all the three replicates, compared with only 64.2 nmol/min/g for controls (Figure 5B). POD activity was also significantly increased compared to controls at 12 hpi, and it peaked at 48 hpi. Again, values for all the three replicates were > 63.8 nmol/min/g, compared with only 55.0 nmol/min/g for the controls (Figure 5C). CAT activity increased over time, values were > 268.2 nmol/min/g for all the three replicates, and they were increased by ∼37.9% compared to the controls (194.5 nmol/min/g; Figure 5D). After infection by Aal for 3 days, DAB and NBT staining showed that PdPap-MsERF105s leaves were lighter brown or blue in color compared to those of controls (Figures 5E,F), indicating that PdPap-MsERF105s leaves contained less ROS than control leaves after pathogen attack.

### Resistance of Transgenic PdPap-MsERF105s Plants to Aal

**FIGURE 6:**
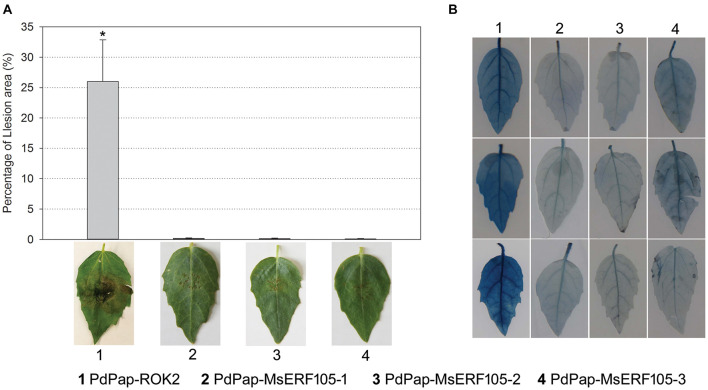
The resistance of PdPap-MsERF105s to Aal. **(A)** The lesion on PdPap-MsERF105s and PdPap-ROK2 after being infected by Aal. **(B)** The cell membrane integrity of PdPap-MsERF105s and PdPap-ROK2 after being infected by Aal. ANOVA was conducted using Duncan’s method, and *p* ≤ 0.05 was considered significant.

To further verify the resistance ability of PdPap-MsERF105 plants, leaves were inoculated with Aal mycelium disks. The results revealed no lesions on the leaves of all the three PdPap-MsERF105s replicates at 5 days postinfection (dpi), but there were large lesions on leaves of the controls at 5 dpi, and the relative lesion area was 26% (Figure 6A). In addition, following infection by Aal for 5 days, Evens blue staining results showed that PdPap-MsERF105s leaves were lighter blue than those of the controls (Figure 6B), indicating that the cell membrane integrity of PdPap-MsERF105s plants was better than that in the controls after pathogen attack.

### Differential Expression of Defense Response Genes in PdPap-MsERF105 Plants

**FIGURE 7:**
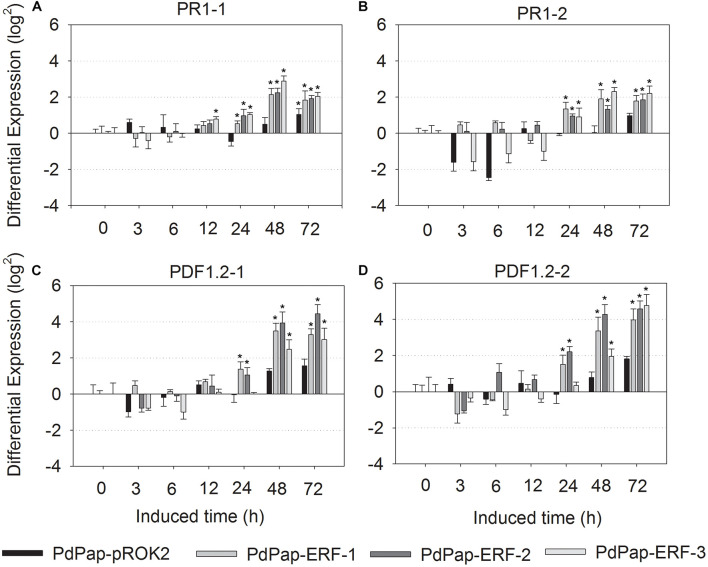
The differential expression of *PR1* and *PDF1.2* in transformant PdPap-MsERF105s. Following infected by Aal, the expression levels of **(A)** *PR1-1*, **(B)** *PR1-2*, **(C)** *PDF1.2-1*, and **(D)** *PDF1.2-2* were determined by RT-qPCR. ANOVA was conducted using Duncan’s method, and *p* ≤ 0.05 was considered significant. RT-qPCR: quantitative real-time PCR.

To explore whether MsERF105 could activate the expression of downstream defense response genes, the expression levels of two *PR1s* (*PR1-1* and *PR1-2*) and two *PDF1.2* proteins (*PDF1.2-1* and *PDF1.2-2*) were determined. The results showed that following infection by Aal, expression levels of *PR1-1* were significantly higher than the controls, with an increase after 12 hpi and a peak at 48 hpi. For Pap-MsERF105-3, the increase was 7.41 (22.89)-folds (Figure 7A). *PR1-2* expression levels were also increased in all the three PdPap-MsERF105s replicates after 24 hpi (Figure 7B). The expression levels of two *PDF1.2* genes were upregulated after 24 hpi, and increased over time, and the expression levels of *PDF1.2-1* were the highest (24.4-folds) in PdPap-MsERF105-2 (increased by 18-fold compared with the control PdPap-ROK2 (Figure 7C). Expression levels of *PDF1.2-2* were the highest (24.8-fold) in PdPap-MsERF105-2, an increase of ∼28-fold compared with the control PdPap-ROK2 (Figure 7D).

## Discussion

*Trichoderma* genus, the widely used biocontrol agents, can induce plant hormone signal pathways, and thereby endow plants with broad-spectrum resistance to phytopathogens (Manigundan et al., 2016). Herein, *M. sieversii* seedlings were treated with *Trichoderma* biofertilizer and/or Aalm, and transcriptomes showed significant differences between treatments. *M. sieversii* seedlings treated with *Trichoderma* biofertilizer and Aalm yielded more DEGs than seedlings treated with either agent alone. We subsequently investigated which genes were differentially expressed and explored their functions.

Gene ontology, COG, and KEGG classifications showed that many DEGs were involved in signal transduction and defense mechanisms. Additionally, following treatment with *Trichoderma* biofertilizer and Aalm, *JAZ* and *MYC2* in the JA signal pathway and *NPR1* in the SA signal pathway displayed significant differential expression. Previous studies also showed that tomato root colonization by *Trichoderma* activated the SA hormone signal pathway to limit nematode root invasion, and it enhanced the JA hormone signal pathway to antagonize the deregulation of JA-dependent immunity against nematodes, and further impeded nematode performance both locally and systemically at multiple stages of parasitism (Medina et al., 2017). Analysis of plant hormones demonstrated that treating with *T. harzianum* T22 before or concurrent with *Cucumber mosaic virus* infection led to systemic resistance through JA/ET and SA signaling pathways in tomato (Antonella et al., 2016). Thus, like pathogens, *Trichoderma* biofertilizer can also activate hormone signaling pathways and defense responses in *M. sieversii*, and thereby protect plants against pathogen attack.

*Trichoderma* biofertilizer activated numerous disease resistance genes and defense response genes. Disease resistance genes included kinase genes (*STK*, *LRR-RLK*, and *MAPK*) and transcription factor genes (*ERF*, *NAC*, *bHLH*, and *HSF*), many of which are responsible for activating downstream defense response genes, such as *DRP*, *ABC*, and *HSP*. Previous studies showed that JA and SA induced increased expression of *OsMSRPK1* (a serine/threonine protein kinase) in *Oryza sativa* (Lee et al., 2006), and overexpression of *Nicotiana repanda NrSTK* (a serine/threonine protein kinase) in the susceptible tobacco variety Honghuadajinyuan, which significantly enhanced resistance to the black shank (Gao et al., 2015). DRP has a predicted N-terminal signal anchor sequence that targets DRP to subcellular membranes or the plasma membrane, but unlike signal peptides, this is not removed by signal peptidase. Plant DRP interacts with pathogen effector proteins, which protect plants from pathogens attack (Takemoto et al., 2012).

Plant ABC transporters play very diverse roles, and some are involved in the transport of defense-related secondary metabolites, defense responses, and cell detoxification, all of which are crucial for plant survival under stress (Devanna et al., 2021). Heat shock proteins (HSPs) are produced in response to stress stimuli. They bind to hydrophobic surfaces of unfolded proteins, preventing their aggregation, and allowing the correct folding of stress-damaged proteins, preventing further cell damage (Piterková et al., 2013). Our current results indicated that *Trichoderma* biofertilizer could strongly induce the expression of resistance genes in *M. sieversii*, and not just genes related to JA and SA signaling pathways.

Among transcription factors, ERF family members were the most differentially expressed, hence we speculated that they are closely related to defense responses. Among ERFs, *MsERF105* was the most significantly differentially expressed. Previous studies showed that ERFs play important roles in regulating plant biotic stress tolerance (Müller and Munné-Bosch, 2015). For example, soybean *GmERF113* was significantly induced by *Phytophthora sojae*, ethylene, and methyl jasmonate, and GmERF113 overexpression caused increased resistance to *P. sojae*, and positively regulated the expression of the PR genes *PR1* and *PR10-1* (Zhao et al., 2017). Overexpression of *Gossypium barbadense GbERFb* in tobacco increased disease resistance to *Verticillium dahliae* (Liu et al., 2017). Thus, *MsERF105* may play an important role in the defense responses of *M. sieversii*.

To explore the functions of *MsERF105* in defense responses, the protein was heterologously expressed in PdPap poplar. Following treatment with Aal, the MDA content was determined, and NBT and DAB staining indicated that the antioxidant ability of transgenic PdPap-MsERF105s plants was superior to that of PdPap-ROK2 plants, presumably because *MsERF105* enhanced the activity of reductases (SOD, POD, and CAT). Similarly, under cold stress, when *Cynodon dactylon* CdERF1 was heterologously expressed in *Arabidopsis* plants, the MDA content was reduced, and the activities of SOD and POD were elevated (Hu et al., 2020). Furthermore, the activities of SOD and POD in ZmERF105-overexpressing lines were markedly higher than in wild-type maize lines after infection with *E. turcicum* (Zang et al., 2020). Thus, *MsERF105* may help to clear ROS after pathogen attack.

Following treatment with Aal mycelia, lesions on PdPap-MsERF105s leaves were significantly smaller than those on PdPap-ROK2 leaves, and cell membrane integrity in PdPap-MsERF105s leaves was better than in PdPap-ROK2 leaves. This suggests that *MsERF105* enhanced the resistance of PaPap poplar to Aal, possibly because *MsERF105* activates the expression of *PR1* and *PDF1.2*. Additionally, overexpression of AcERF2 induced the accumulation of transcripts of plant defense-related genes (PR1, PR2, and PR5), and increased Arabidopsis resistance to the pathogens *Pseudomonas syringae* pv. tomato DC3000 and *B. cinerea* (Sun et al., 2018). *Overexpression of ZmERF105* in soybean enhanced the expression of several PR genes, including *ZmPR1a*, *ZmPR2*, *ZmPR5*, *ZmPR10.1*, and *ZmPR10.2*, following infection with *E. turcicum* (Zang et al., 2020). These results indicate that *MsERF105* plays a positive modulatory role in response to pathogen infection in *M. sieversii*.

In conclusion, *Trichoderma* biofertilizer not only activated JA and SA signaling pathways but also induced the differential expression of numerous disease resistance genes and defense response genes, especially ERF transcription factor family members. Heterologous expression of *MsERF105* significantly enhanced the antioxidant and antipathogen abilities of transgenic PdPap poplar. The findings suggest that *MsERF105* is crucial for the response to pathogen attack in *M. sieversii*.

## Data Availability Statement

The original contributions presented in the study are publicly available. The raw transcriptome data have been deposited in the SRA database. The accession numbers of Con transcriptome data are SRR16148375, SRR16148374, and SRR16148363, the accession numbers of T6 transcriptome data are SRR16148361, SRR16148360, and SRR16148359, the accession numbers of A6 transcriptome data are SRR16148358, SRR16148357, and SRR16148356, the accession numbers of TA6 transcriptome data are SRR16148373, SRR16148372, and SRR16148355, the accession numbers of T48 transcriptome data are SRR16148371, SRR16148370, and SRR16148369, the accession numbers of A48 transcriptome data are SRR16148368, SRR16148367, and SRR16148366, and the accession numbers of TA48 transcriptome data are SRR16148365, SRR16148364, and SRR16148362. And the transcriptome assembly data have been deposited in the SRA database, and the accession number is GJMU 00000000. BioProject number is PRJNA 736527, and BioSample number is SAMN 19651934.

## Author Contributions

SJ: conceptualization, formal analysis, investigation, methodology, and roles/writing – original draft. ZL: conceptualization, data curation, formal analysis, funding acquisition, methodology, supervision, and writing – review and editing. YW: conceptualization, data curation, funding acquisition, methodology, project administration, supervision, and writing – review and editing. All authors contributed to the article and approved the submitted version.

## Conflict of Interest

The authors declare that the research was conducted in the absence of any commercial or financial relationships that could be construed as a potential conflict of interest.

## Publisher’s Note

All claims expressed in this article are solely those of the authors and do not necessarily represent those of their affiliated organizations, or those of the publisher, the editors and the reviewers. Any product that may be evaluated in this article, or claim that may be made by its manufacturer, is not guaranteed or endorsed by the publisher.

## Supplementary Material

Supplementary Figure 1 Poplar transformation PdPap-MsERF105s construction. **(a)** Resistant buds of transformant PdPap-MsERF105s; **(b)** asexual reproduction of PdPap-MsERF105s; and **(c)** transcription detection of MsERF105 gene in transformant PdPap-MsERF105s by RT-qPCR. The data were counted by RT-qPCR, ANOVA was conducted using Duncan’s method, and *p* ≤ 0.05 was considered significant. RT-qPCR: quantitative real-time PCR.
