# Supplementary figures and images for: Trichoderma-Induced Ethylene Responsive Factor MsERF105 Mediates Defense Responses in Malus sieversii
Source: Front Plant Sci. 2021 Oct 29;12:708010. doi: 10.3389/fpls.2021.708010 (PMC8585786; doi:10.3389/fpls.2021.708010)

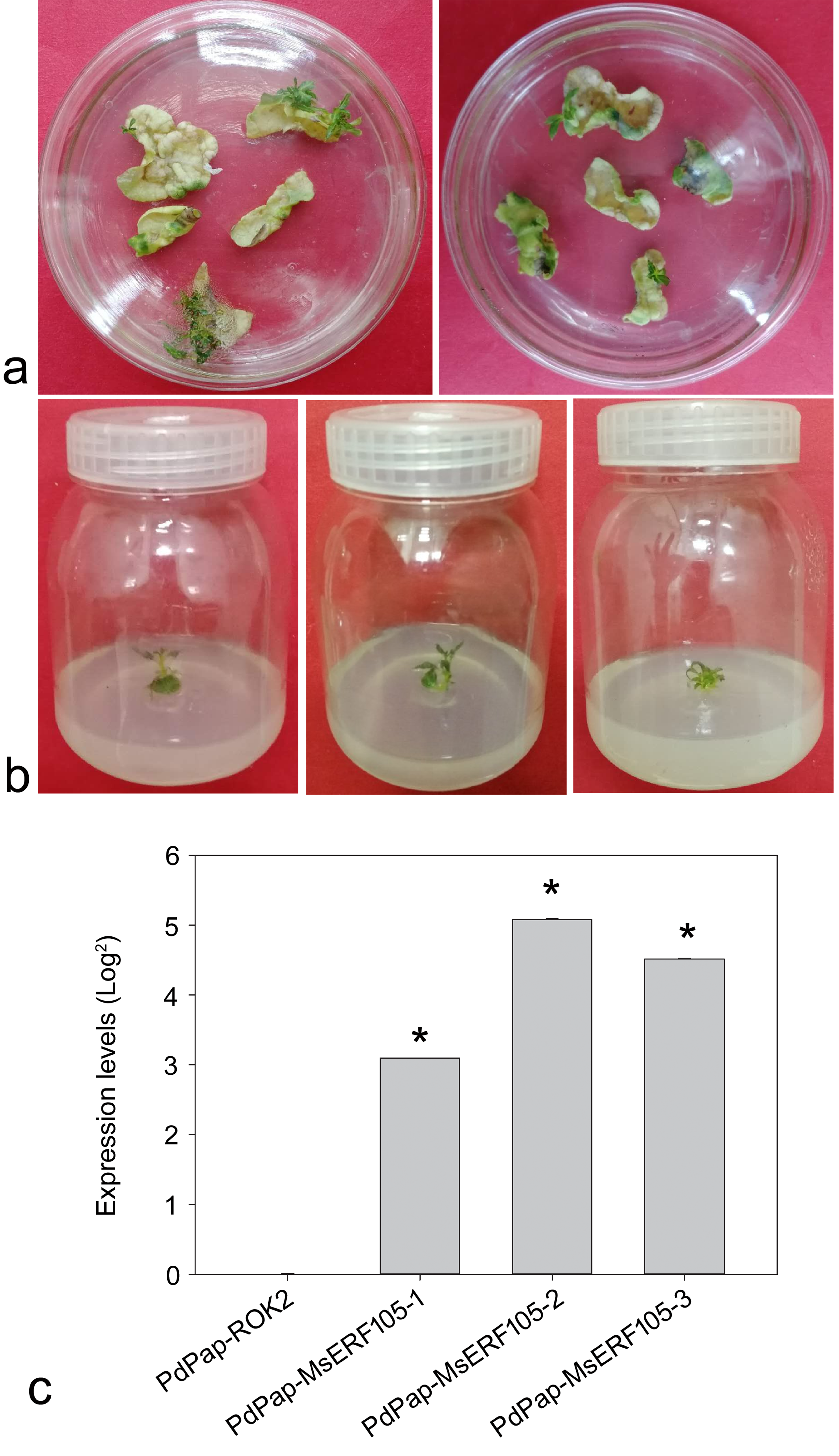

Supplement: Supplementary Figure 1 — Poplar transformation PdPap-MsERF105s construction. (a) Resistant buds of transformant PdPap-MsERF105s; (b) asexual reproduction of PdPap-MsERF105s; and (c) transcription detection of MsERF105 gene in transformant PdPap-MsERF105s by RT-qPCR. The data were counted by RT-qPCR, ANOVA was conducted using Duncan’s method, and p ≤ 0.05 was considered significant. RT-qPCR: quantitative real-time PCR. [file Image_1.TIF]
