# Supplementary material for: Trichoderma-Induced Ethylene Responsive Factor MsERF105 Mediates Defense Responses in Malus sieversii
Source: Front Plant Sci. 2021 Oct 29;12:708010. doi: 10.3389/fpls.2021.708010 (PMC8585786; doi:10.3389/fpls.2021.708010)
Supplement: Supplementary file 2 [file Table_1.DOCX]

**Supplemental Table 1** Primers used for qRT-PCR.

| Gene name | Primer name | Primer sequence (5'---3') | Tm (℃) |
| --- | --- | --- | --- |
| *MsERF61* | MsERF61L | AGGGAGTCTGATCTCGGCTACC | 58.8 |
|  | MsERF61R | CCAGCAGCGGCAGTTCTGTAT | 58.9 |
| *MsERF105* | MsERF105L | CCTTCGACACCGCCGTTGAA | 59.0 |
|  | MsERF105R | CATCTCCTGAACATCCTCCTCTTTCTT | 59.2 |
| *MsERF17* | MsERF17L | GGGAGCGAATCTGGTTGGGATC | 59.1 |
|  | MsERF17R | ATCGACTGAGACTCCGCCTGAA | 59.2 |
| *MsERF4-1* | MsERF4-1L | GTTCAGATCGACCACCGAGGAC | 58.8 |
|  | MsERF4-1R | CCTTCCAGCACCATCAGGTTCC | 59.2 |
| *MsERF112* | MsERF112L | CGAGGAATCGACCATCAGAGCC | 58.9 |
|  | MsERF112R | CGACAACGCCGCCAGAGATT | 59.1 |
| *MsERF026* | MsERF026L | TATACGGTCTGCGGCGGCTA | 58.9 |
|  | MsERF026R | AATCCTCGGCGGACTCACCA | 59.0 |
| *MsERF060* | MsERF060L | CGATGCGAAGCTCCAGGAGATT | 58.9 |
|  | MsERF060R | CGGATTCCGGCGTAGATGACTC | 59.0 |
| *MsERF1A* | MsERF1AL | CCATCCCTGCTCCCATTTCCAA | 58.6 |
|  | MsERF1AR | GACGCGGCTCTTGCTTACGA | 58.9 |
| *MsERF023* | MsERF023L | GGAGCAACCACCGTACACTGAA | 58.9 |
|  | MsERF023R | TTGAGGCAGTAGGCGGCAAC | 58.7 |
| *MsERF4-2* | MsERF4-2L | TACGCCGCCGAGATCAGAGAT | 59.0 |
|  | MsERF4-2R | GCTGCTGGGACTGTTGCTGA | 58.6 |
| *MsERF SHINE3* | MsERF SHINE3L | CAGAGGCAGCAGCAAGAGCATA | 59.0 |
|  | MsERF SHINE3R | TGTCGAGTCGAAGGCAAGTCAG | 58.7 |
| *AUX/IAA* | C23695-L | AAGTTAGCATGGATGGAGCACCTT | 58.8 |
|  | C23695-L | CTCTTCACAGGACCAAGCAGCAT | 59.3 |
|  | C36501-L | AGTGGTTGGCAAACAGTTCATCATC | 58.9 |
|  | C36501-R | CGAAGTTTCATTTGGTGACTCCGAAT | 59.1 |
|  | C18973-L | CCCATAGATGTCCCTCCCACCA | 58.9 |
|  | C18973-R | TCCAGTCGCCGTCTTTGTCTTC | 58.9 |
| *JAZ* | C40102-L | TGCTGCTGCTGCTGATAAGAAGA | 58.8 |
|  | C40102-R | AAGTAGGATATGGACTGTTGCTGACC | 59.1 |
|  | C33528-L | ATCCTCCAAATCCCAGTTCCAGAAAT | 59.0 |
|  | C33528-R | AGCAGCAACCGTGAAAGAAGAAAC | 59.0 |
|  | C43172-L | CCATGACGGCTGATTCTAGTGTGA | 59.0 |
|  | C43172-R | CGGAGGAACTGAACTGCTGGAAT | 59.0 |
| *MYC2* | C19865-L | AAGTGAAGGACCCGGTGAATGC | 59.2 |
|  | C19865-R | GCTGCTCCTGTCATATCCGTTGTA | 59.0 |
|  | C40416-L | GGTGGTTAGGGAGGCGGATAGTA | 59.1 |
|  | C40416-R | TCGTTGATGTAGGATATGGCGTCTC | 59.0 |
|  | C38512-L | ACTTCTCCTTCGCCGACATTGG | 59.0 |
|  | C38512-R | CTCTCCGCTTCCACATGGTTCAA | 59.3 |
| *NPR1* | C36229-L | GAAGAGCACCTTAGCCGTCTACAA | 58.9 |
|  | C36229-R | TCCTCCTTGTCCTTGTCGAATGC | 59.0 |
| *MsActin* | MsActinL | TTCATTCACCACCACAGCAGAG | 58.9 |
|  | MsActinR | CCAGCAGCCTCCATTCCGAT | 58.9 |
| *PdPR1-1* | PdPR1-1L | CCTCCACCGCAACTCACTTCTT | 58.8 |
|  | PdPR1-1R | CCAGCAGTGAAGAGGGAAACGA | 58.6 |
| *PdPR1-2* | PdPR1-2L | ACCACCGTGCAAGCCTATGC | 59.1 |
|  | PdPR1-2R | AGAGTTACGCCAAACCACCTGAG | 59.0 |
| *PdPDF1.2-1* | PdPDF1.2-1L | CTGCTCCTAGTCTTGGCTTCTCA | 58.0 |
|  | PdPDF1.2-1R | AAGAGCCTCGCAGCATCTACATAT | 58.3 |
| *PdPDF1.2-2* | PdPDF1.2-2L | TCCACATATAACGGCGGAAGTCTTT | 59.0 |
|  | PdPDF1.2-2R | CGGGATTAATTGACTCGGAACTTGAC | 59.0 |
| *PdActin* | PdActinL | TTCCGTTGCCCTGAGGTCCTAT | 59.1 |
|  | PdActinR | TCAGGAGGAGCAACCACCTTGA | 59.3 |
